# Hand contour detection in wearable camera video using an adaptive histogram region of interest

**DOI:** 10.1186/1743-0003-10-114

**Published:** 2013-12-19

**Authors:** José Zariffa, Milos R Popovic

**Affiliations:** 1 Toronto Rehabilitation Institute, University Health Network, 550 University Avenue, #12-102, Toronto M5G 2A2, Ontario, Canada; 2 Institute of Biomaterials and Biomedical Engineering, University of Toronto, Toronto, Canada

**Keywords:** Hand contour, Wearable system, Colour histogram, Hand function, Neuro rehabilitation

## Abstract

**Background:**

Monitoring hand function at home is needed to better evaluate the effectiveness of rehabilitation interventions. Our objective is to develop wearable computer vision systems for hand function monitoring. The specific aim of this study is to develop an algorithm that can identify hand contours in video from a wearable camera that records the user’s point of view, without the need for markers.

**Methods:**

The two-step image processing approach for each frame consists of: (1) Detecting a hand in the image, and choosing one seed point that lies within the hand. This step is based on *a priori* models of skin colour. (2) Identifying the contour of the region containing the seed point. This is accomplished by adaptively determining, for each frame, the region within a colour histogram that corresponds to hand colours, and backprojecting the image using the reduced histogram.

**Results:**

In four test videos relevant to activities of daily living, the hand detector classification accuracy was 88.3%. The contour detection results were compared to manually traced contours in 97 test frames, and the median F-score was 0.86.

**Conclusion:**

This algorithm will form the basis for a wearable computer-vision system that can monitor and log the interactions of the hand with its environment.

## Introduction

Hand function is essential to most activities of everyday life. Injuries to the nervous system, for example stroke or spinal cord injury (SCI), can severely impair hand function and thus reduce the affected individual’s independence and quality of life. In order to minimize these negative consequences and ensure that as much function as possible is regained, an intensive rehabilitation process is undertaken following injury. Despite current best practices in rehabilitation, however, the recovery of hand function remains the top priority of individuals with tetraplegia [1]. The search for new interventions to enhance functional recovery after neurological injury is thus ongoing, and includes pharmacological interventions [2], physical and occupational therapy approaches [3], and medical devices such as functional electrical stimulation systems [4-6] or rehabilitation robotics [7,8].

Regardless of the nature of the intervention being investigated, tracking the amount of recovery over time is crucial to determining the effectiveness of the novel approach. Various clinical assessments exist to measure hand function, for example the Graded and Redefined Assessment of Strength, Sensibility and Prehension (GRASSP, [9]), the Action Research Arm Test (ARAT, [10]), the Jebsen hand function test [11], the Sollerman hand function test [12], the grasp-and-release test [13], and the Toronto Rehabilitation Institute Hand Function Test (TRI-HFT, [14]). Without exception, these tests are designed to be administered in a laboratory or clinical setting by trained personnel, and are typically not performed more frequently than once every few weeks. Furthermore, performance in these settings is not necessarily reflective of how much an individual is actually using his or hand during daily life. Methods to assess hand function on a continuous basis at home and in the community are lacking. The use of wearable sensors in rehabilitation has been actively investigated in recent years with applications including the remote monitoring of lower limb function and fall detection [15], but the sensors typically employed in these studies (e.g., accelerometers) are too simple to capture the very complex behaviour of the human hand.

We propose to use a computer vision system based on unobtrusive wearable cameras to monitor hand function at home. Specifically, we plan to develop a system capable of detecting interactions of the hand with objects in the environment, and quantifying these interactions using metrics such as the number of grasp attempts (which is indicative of how much a subject is relying on attendant care) and the number of dropped objects. This log of at-home hand use can then be provided to a clinician to help refine an individual’s outpatient rehabilitation program or to evaluate the success of a new intervention.

The objective of the study presented here was to develop the first step of such a system, which is to correctly locate and segment the hand. Because the hand and the objects that it is interacting with are in very close proximity or overlapping, an accurate estimate of the hand outline in the image is essential to correctly detecting manipulation in the video.

Many hand tracking systems have been proposed in the context of gesture recognition for human-computer interaction [16-23]. These approaches typically fall into one of two categories: methods that are trained to recognize a pre-defined set of poses based on their appearance, and model-based methods that attempt to fit an articulated model of the hand to the image [23,24]. Model-based methods are more flexible, but more computationally demanding. Researchers have proposed a variety of formulations of the optimization problem required to fit a model to an image, in order to achieve both high accuracy and computational efficiency [19,25-28]. Work is ongoing in this area and, more recently, the use of depth sensing in combination with colour information has been explored to improve model-based hand tracking performance [23,29]. Regardless of the approach used, however, these systems rarely need to deal with the varying backgrounds and moving camera inherent in a wearable system. Conversely, wearable systems either have been restricted to recognizing pre-defined hand postures [30,31] or have not provided the contour of the hand [32-34], whereas this information is important in a rehabilitation context. That said, processing of egocentric video is an area currently attracting a lot of research interest, and the technologies being developed to analyze and record daily activities may increasingly provide tools applicable to neurorehabilitation [35-37], though no studies have explicitly focused on this area as of yet. Because our system needs to be deployable in a clinical population, it should be aesthetically acceptable to an average person, and ideally based on low-cost commercially available components. In contrast to previous studies, our system requirements were therefore as follows: (1) identify the contour of the hand against an arbitrary and changing background without the need for markers, (2) use video from a single wearable camera, and (3) impose no constraints on hand posture. We propose a novel, simple and flexible colour-based solution to hand contour detection that satisfies these requirements.

## Methods

### System overview

The hardware component of the system consists of one Looxcie 2 wearable camera (Looxcie Inc., USA). This small camera, similar to a Bluetooth headset in size and shape, is worn over the ear and records video from the user’s point of view. Video was recorded at 27 frames per second with a resolution of 640 × 480 in mp4 format.

The image processing algorithm consists of two stages, which are applied to each frame individually. The first stage is responsible for determining whether or not a hand is present in the image, and identifying a single point that lies within the hand. This task can be accomplished much more robustly than detecting all points belonging to the hand. The chosen point is then used as the seed point for the second stage, which is responsible for detailing the contour of the region containing the seed point (i.e., the hand). All image processing was conducted in C++ using the OpenCV libraries [38].

### Hand detection and seed point identification

The first stage of the image processing algorithm is simultaneously responsible for determining whether or not a hand is present in the frame, and for selecting a seed point that lies within the hand. In order to accomplish this, every region of the image that could potentially represent a hand was first identified. This determination was based on colour. The image was first back-projected using a histogram obtained from the mixture-of-Gaussians skin model of Jones and Rehg [39] (30 Hue bins, 32 Saturation bins, and 32 Value bins). If the highest value in the backprojection was below a threshold, the image was judged to contain no skin regions and therefore no hand, and no further processing was performed. Otherwise, all pixels in the resulting image that were less than 0.5 times the highest value found in the backprojection were set to 0. The resulting skin image indicated what regions of the frame potentially corresponded to a naked hand.

Following morphological operations (dilation followed by erosion) to remove small gaps, contour detection was performed on the skin image. The resulting contours were judged on two criteria: size and texture. Any contour whose area was less than 1% of the total frame area was eliminated. For texture, the Laplacian of the grayscale image inside each contour was computed and normalized by the area of the contour, then compared to a threshold. Contours with texture values above the threshold were eliminated. The rationale for this procedure was that the skin of the hand is expected to be relatively smooth, and the lack of local variations will result in a low Laplacian value. In contrast, objects with more local variations will have higher values. This comparison is useful to eliminate objects that may be very similar to skin in terms of colour, but have a different visual texture, in particular wood.

After all contours that did not meet the size and texture thresholds had been eliminated, the following determination was made. If there were no contours left, the image was judged not to contain a hand. If there was a single contour left, it was taken to be the hand. If there were exactly two contours left, the right-most one was selected (this is useful in cases where both hands are visible in the frame; the right-hand was selected here as the default target). If there were more than two contours left, the one with the largest area was taken to be the hand.

**Figure 1:**
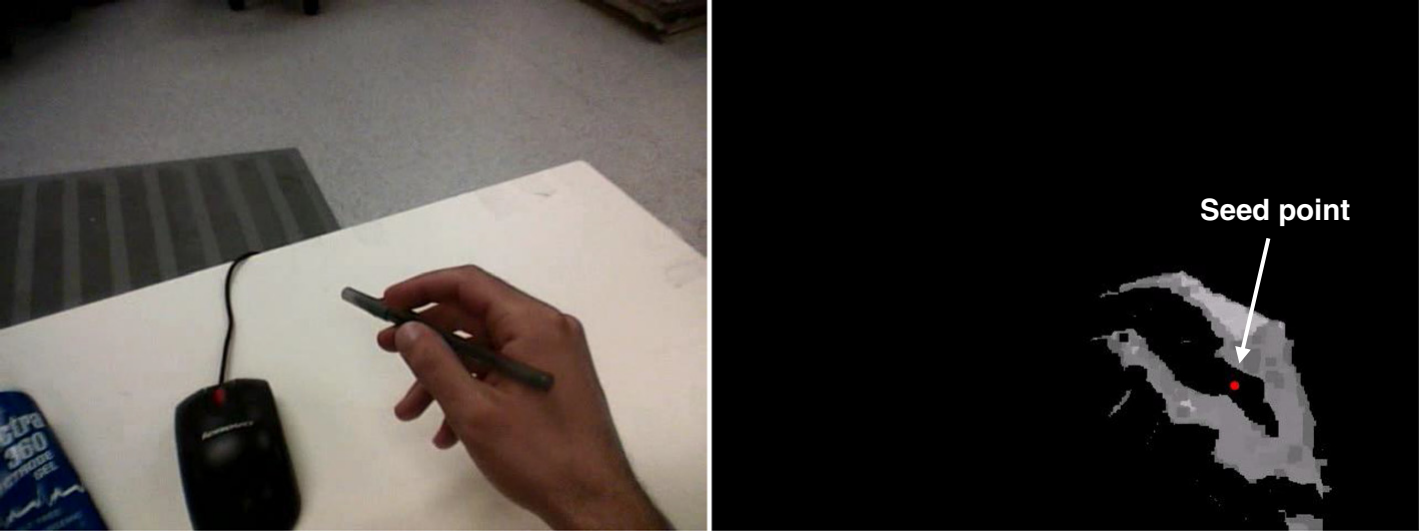
**Illustration of the seed point detection step.** The original image (left) is converted to a skin image, to which thresholding and morphological operations are applied (right). Once a hand has been identified, the seed point is the centroid of the filled hand contour.

Once the hand contour had been detected, the contour was filled and its centroid was selected as the seed point. The hand detection and seed point determination process is illustrated in Figure 1.

### Hand contour determination

Our identification of the hand contour relies primarily on identifying the region in the image’s colour histogram that corresponds to hand pixel colours. For this purpose, the image was first converted to a Hue-Saturation-Value (HSV) colour space. The Hue dimension was circularly shifted by one third of the total Hue range. This operation helped reduce the instability that can occur in poor lighting conditions as a result of the skin Hue values being close to a discontinuity in the Hue distribution (0°/360° point) [40]. A coarse colour histogram was then built using the Hue and Saturation dimensions. The Value dimension was ignored in order to reduce sensitivity to lighting variations. The histogram discretized the space into 20 Hue bins and 16 Saturation bins.

The histogram bin corresponding to the seed point selected above was identified. A gradient ascent was then conducted in the neighbourhood of this point to identify the corresponding local maximum in the histogram. A region of interest (ROI) in the histogram was then selected using a two-step process. First, a rectangular region was selected around the chosen local maximum. Let h and s be the hue and saturation indices, respectively, *H*(h,s) be the histogram function, and *H*(h_max_,s_max_) be the value of the local histogram maximum. The rectangular region was defined by finding the largest possible entries of the quadruplet (k_1_,k_2_,k_3_,k_4_) satisfying the conditions in Equation 1.

(1) $$\begin{array}{c} \left\{k_{1}\left|\forall \;k\;\leq \;k_{1},H\left(h_{\max}+\;k,s_{\max}\right)>H\left(h_{\max},\;s_{\max}\right)\times \alpha_{1}\right)\right\}\\ \left\{k_{2}\left|\forall \right)\;k\;\leq \;k_{2},H\left(h_{\max}\;-\;k,\;s_{\max}\right)>H\left(h_{\max},\;s_{\max}\right)\times \alpha_{2}\right\}\\ \left\{k_{3}\left|\forall \;k\;\leq \;k_{3},\;H\left(h_{\max},s_{\max}+k\right)>\;H\left(h_{\max},\;s_{\max}\right)\times \alpha_{3}\right)\right\}\\ \left\{k_{4}\left|\forall \;k\;\leq \;k_{4},\;H\left(h_{\max},s_{\max}\;-\;k\right)>H\left(h_{\max},\;s_{\max}\right)\times \alpha_{4}\right)\right\} \end{array}$$

We empirically set α_1_, α_2,_ α_3_ and α_4_ to 0.05. All histogram bins outside of this rectangular region were set to 0. The second step of the ROI selection process consisted of applying a flood-fill algorithm to the histogram, using the local maximum as the starting point. The rule for adding a new bin to the filled region was that its value be smaller than or equal to the value of a neighbouring bin already belonging to the filled region. This process made it possible to select an irregularly shaped region associated with a single local maximum in the histogram. The resulting region was assumed to correspond to the space of hand colours.

**Figure 2:**
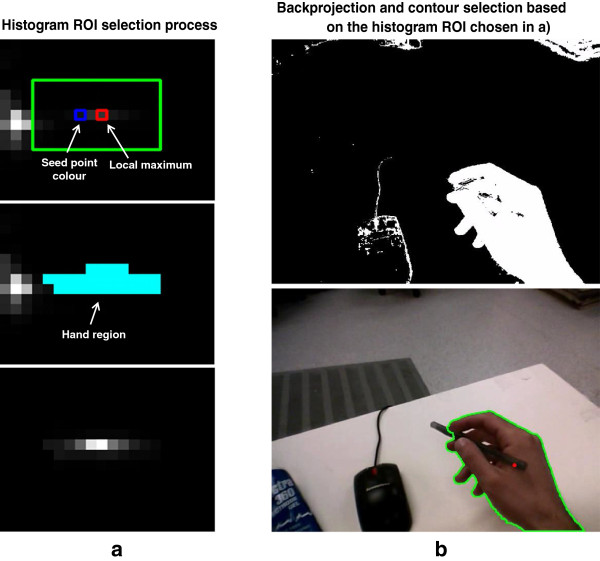
**Illustration of the hand contour determination process. a)** Selection of the histogram region corresponding to the hand. A rectangular region is first identified in the Hue-Saturation histogram, based on the local maximum closest to the seed point colour (top). A flood-fill algorithm is used to refine the histogram ROI (middle), and all bins outside of this region are set to 0 (bottom). **b)** Backprojection of the HSV image using the reduced histogram shown at the bottom of a) (top), and resulting hand contour superimposed on the original image (bottom).

All histogram bins outside of the ROI were set to 0, and the resulting histogram was used to backproject the HSV image. The result was converted to a binary image and provided an image identifying skin regions. Since this skin image is obtained adaptively using information from the video frame itself, it is much more precise that the skin image obtained in the first step using standard skin colour models [39] that are not tailored to this particular image. Lastly, a contour identification process was applied to the skin image, and the contour containing the seed point was chosen as the hand contour. If no contours contained the seed point, no hand was detected. The hand contour detection process is illustrated in Figure 2.

### Evaluation of the system

Four 30-second test videos were recorded from a single subject to evaluate the system. The subject consented to participate in the study. The content of the videos was designed to be representative of common everyday situations, and was as follows:

1. Single hand interacting with objects of different shapes (mouse, pen, credit card, tube) on a desk in an office environment.

2. Single hand interacting with similar objects as in the first video (mouse, pen, tube, mobile phone), but with the addition of more cluttered backgrounds, including another person in the background.

3. Use of a toothbrush in a bathroom environment. Both hands are visible in certain frames.

4. Use of a spoon to eat cereal. Both hands are visible in certain frames.

To evaluate the hand detection component of the system, the frames containing a hand were manually determined and compared to the results of the automated system. The overall accuracy, sensitivity, specificity, positive predictive value, and negative predictive value of the classifier were computed.

To evaluate the contour identification component of the system, hand contours were traced manually, and the resulting traces were used as the ground truth to which the system output was compared. Because tracing every frame was prohibitive, the first frame of each second of video was selected (i.e., 31 frames per video) and, if it contained a hand, the contour of the hand was traced. Two metrics were then used. First, letting A be the hand region identified by the automated system, and M be the manually traced hand region, the error for a given frame was quantified as shown in Equation 2 [41].

(2) $$\mathrm{Err}=\frac{\text{Area}\left(A\;\cup \;M\right)\;-\;\text{Area}\left(A\;\cap \;M\right)}{\text{Area}\left(M\right)}\times 100$$

The second metric used was the F-measure, which is defined as the harmonic mean of precision and recall. In this situation, these are the pixel-wise precision and recall calculated based on a pixel’s inclusion in A and/or M [37].

## Results

### Hand detection results

The four test videos contained a total of 3297 frames. Of these, 2911 contained at least one hand. The results of the 2-category classifier for hand detection (hand present vs. no hand present) are given in Table 1 for each of the test videos separately. For all videos combined, the overall classification accuracy was 88.3%.

**Table 1:** Performance of the hand detection classifier

**Video**	**Overall accuracy**	**Sensitivity**	**Specificity**	**Positive predictive value**	**Negative predictive value**
1	89.16%	88.58%	95.59%	99.56%	42.76%
2	88.17%	87.57%	91.96%	98.57%	53.93%
3	83.58%	81.79%	91.78%	97.86%	52.34%
4	92.42%	92.22%	95.00%	99.58%	48.72%

### Contour determination results

**Figure 3:**
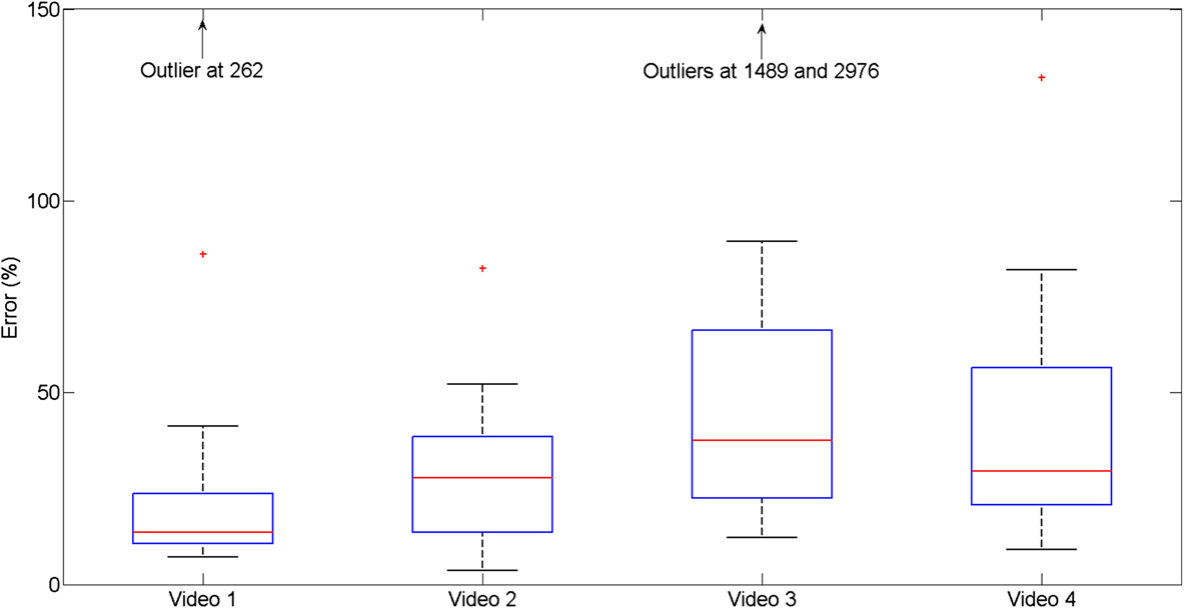
**Distribution of errors for each of the four test videos.** The error is computed as defined in Equation 2.

**Figure 4:**
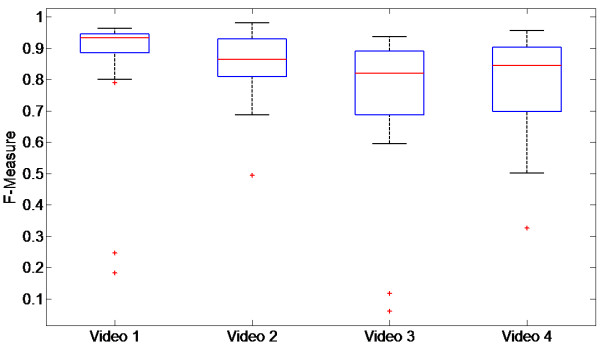
Distribution of F-scores for each of the four test videos.

After the hand detection classifier was applied to the 124 test frames (1 per second in each video), of which 106 actually contained a hand image, the number of frames considered to contain a hand was 25, 24, 21, and 27, respectively, for each of the four test videos. Thus, the contour detection algorithm was applied to a total of 97 test frames. The error distribution for each of the test videos is provided in Figure 3. The overall median error for all 97 frames was 26.8%. The F-scores for each test video are provided in Figure 4, and the overall median F-score for all 97 frames was 0.86.

**Figure 5:**
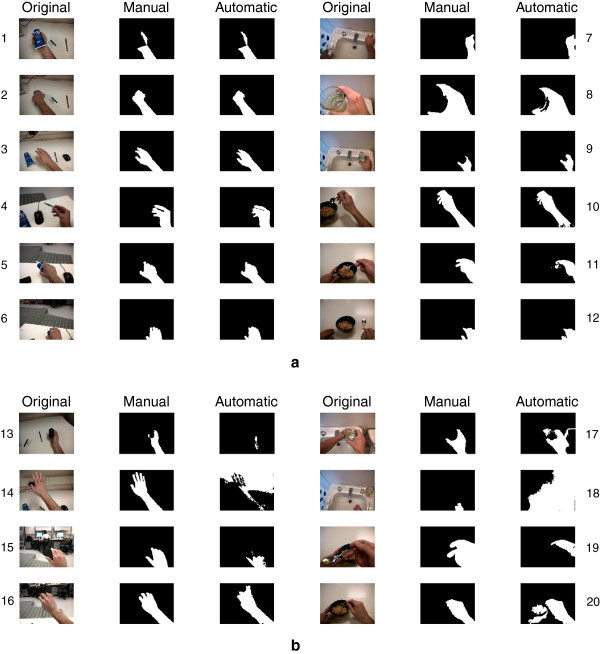
**Examples of the system output.** In each case, the original video frame is shown on the left, the manually traced hand contour is provided in the middle, and the output of the automated hand contour is provided on the right. **a)** Examples of situations where good performance was achieved. **b)** Examples of situations in which further improvement is needed (refer to the text for discussion).

**Figure 6:**
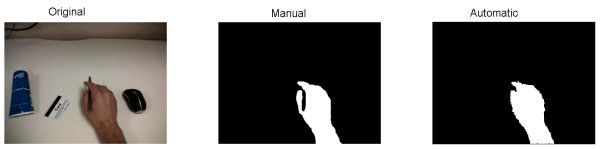
Example of a frame for which the error level (26.6%) was representative of the median error over all test frames (26.8%).

To provide more insight into the performance achieved, Figure 5 shows a number of representative frames illustrating both scenarios with good performance and scenarios highlighting areas for improvement. In each case, the original image is shown, along with the manually traced hand contour and the automatically detected hand contour. In addition, Figure 6 illustrates a frame for which the error was 26.6%, and which was therefore representative of the median error.

### Speed of execution

When evaluated on the pre-recorded test videos, the entire image processing algorithm (comprising both hand detection and contour determination) executed at a speed of approximately 15.1 frames per second on a desktop PC (Intel Core i3, 3.3 GHz, 4 GB RAM).

## Discussion

We have developed an image processing approach capable of detecting and segmenting the hand in video obtained from a wearable camera. Our approach relies on a novel adaptive histogram ROI selection, and requires neither markers nor constraints on the hand posture. The resulting algorithm is the first step in a planned wearable system for monitoring hand function outside of the laboratory or clinical environment during the course of neurorehabilitation. To the best of the authors’ knowledge, no other fully automated hand tracking system is available that meets all of the system requirements outlined in our introduction. A close attempt is the system proposed by Fathi et al. [35], which does include a segmentation of the hands in egocentric video, but is focused on object recognition and is based on a training set of different activities all taking place in the same specific scene (i.e., the camera moves with the user’s head, but the background panorama is not varying). Since the method constructs a model of the background, it is unclear how well suited the algorithm would be to dealing with more variable backgrounds in a fully automated way. Li and Kitani [33] very recently proposed a method that would be appropriate for our application, that relied on selecting among a set of hand detectors depending on the global illumination conditions, but neither the implementation nor the training set used to create the models are publicly available at the time of writing. In the absence of appropriate comparison methods, we evaluated the performance of our method by comparing it to a “ground truth” of manually traced outlines. The F-measure was used in both our study and the one by Li and Kitani, and can therefore provide some degree of performance comparison, albeit on different datasets. Li and Kitani reported a performance of 0.84 on their testing set in the best case, which is closely comparable to the 0.86 value reported here. Given that their testing set was more extensive than ours, it is possible that their method might have more robust performance across a variety of usage scenarios, but further testing will be required to ascertain this.

Currently the proposed approach operates on a frame-by-frame basis. This is beneficial in the context of the hand contour detection, because the configuration of the hand is highly complex and can change completely in a very short amount of time, making useful modeling of hand state transitions across several frames extremely difficult. Similarly, our method does not require any parametric modeling of the hand anatomy. This independence from both temporal and anatomical modeling is designed to improve the robustness of the algorithm. The frame-by-frame adaptive nature of the contour detection algorithm also reduces the sensitivity to changes in lighting conditions. On the other hand, the hand detection step could likely be improved by taking into consideration several consecutive frames and ensuring some temporal smoothness in the detection results. This will be explored in future versions of the system.

Even without taking into account several frames simultaneously, the performance on four test videos demonstrated good ability to detect the presence of the hand in the image, with an overall classification accuracy of 88.3%. The low negative predictive values in Table 1 indicate that the most common misclassifications were in situations in which a hand was present but was not detected. This occurs primarily when the hand occupies only a small portion of the image, for example if only a portion of the hand is visible at the edge of the frame.

The performance in the test videos also demonstrated that the system provides a useful estimate of the hand contour. This is reflected both by the mean errors and F-scores in Figures 3 and 4 and by the example frames in Figure 5a. Nonetheless, areas for improvement remain in the contour detection, and are illustrated by representative cases in Figure 5b. In certain frames, the hand area can be either underestimated (examples 13, 15, 19 in Figure 5b) or overestimated (examples 16, 17, 20). These cases respectively occur when the histogram ROI selection process (Figure 2) misses bins that correspond to hand pixel, or erroneously includes bins that do not correspond to hand pixels. The likelihood of these two scenarios depends on the coarseness of the histogram: as the histogram becomes more fine-grained, it is more likely that hand pixels will be missed, but less likely that non-hand pixels will be included. Conversely, a coarser histogram is less likely to omit hand pixels but more likely to include non-hand pixels. Underestimation of the hand is more likely if there are large variations in lighting within the hand region (example 19), such that the hand pixels are spread into two distinct regions of the histogram. The stopping criteria for the flood-fill algorithm also play a role in determining the ROI and thus regulating the sensitivity of the system. Lastly, if an incorrect local maximum is selected during the search around the seed point bin, a background region may be erroneously chosen as the hand contour (examples 14, 18). A similar result could occur if an incorrect seed point was chosen. A limitation of the current study is that the contour detection performance could not be evaluated on every frame in the test videos, because manually tracing the hand outline for all frames would have been prohibitive. We must therefore assume that the 97 evaluation frames that were selected at regular intervals in the videos are representative of the overall performance.

The 15.1 frames per second (fps) rate achieved is sufficient for our target application. Although it was not fast enough to process the 27 fps test videos in real time, the system is intended to monitor interactions of the hand with its environment in the context of neurorehabilitation. The hand activities that will need to be monitored will occur over periods of several seconds, and typical scenarios will not require the precise tracking of very fast hand movements. 27 fps is therefore a faster rate than would actually be necessary, and 15 fps is expected to be more than sufficient. Continuing improvements in processor speeds will also increase the number of frames that can be processed in real-time in the near future.

The major limitation of the system in its current form is a degradation in performance in situations where large sections of the background have similar colours to the hand (e.g. wood or beige walls). The use of texture was explored as a way to avoid selecting the wrong region of the image altogether (see Methods), but this does not prevent the contours of the hand from being overestimated if the hand is positioned over a background of very similar colour. Similarly, scenes with cluttered backgrounds result in more complex colour histograms and make it more difficult to clearly identify the range of hand pixel colours. These difficulties are common limitations in primarily colour-based algorithms for skin detection. A possible solution to this problem for our proposed method would be to explore varying the histogram coarseness on a frame-by-frame basis, to provide finer-grained colour discrimination in situations where the hand does not contrast strongly with its environment. Colour normalization schemes or the use of alternative colour spaces may also have an influence on performance. These variations have been left to explore in future work. Note also that the types of background that will prove challenging in this respect will depend on the skin colour, so it is important that the system be able to adapt to those variations. In the present work, different skin colours were not investigated, but are not expected to degrade performance, for two reasons. First, the hand detection step was based on skin colour models constructed from image databases containing various skin colours, and therefore expected to be robust in the presence of such variations [39]. Second, the contour step is based on a dynamic analysis of the colour histogram at each frame, and has no predefined expectations as to what the skin colour should be. The system currently does not support situations in which the hand is covered by a glove, but this could be remedied in the future by using alternate colour models in the hand detection step, tailored to the expected colour of the glove.

Our dataset for this study was limited in size, primarily because manually tracing the hand outlines is a labour-intensive process. The advantage of this approach is that it allowed us to precisely quantify the performance of our approach rather than relying on qualitative evaluations, but the drawback is that our parameter choices may not generalize as well as what could have been obtained with a larger dataset. The parameters in this study were determined by trial-and-error, because the performance of each stage of the algorithm is dependent on several parameters, and optimizing the process would have required a detailed sensitivity analysis that is beyond the scope of this initial investigation (for example, the performance of the hand detection classifier is affected by a set of parameters including the threshold applied to the initial backprojection, the size threshold applied to the regions in the resulting binary image, and the texture threshold applied to the Laplacian operation). Therefore, further data collection and parameter refinement would be advisable in the future, but we expect that the basic method will remain viable. We further expect that a set of parameters can be selected that will yield good performance in all situations, i.e., that there will be no need for case-by-case tuning of parameters and that the method will remain fully automated. This expectation is reasonable for two reasons: (1) Our method is designed specifically to adapt to variations in skin colour, which is the feature that is expected to vary the most between different subjects and different environmental and lighting conditions. (2) Minor fluctuations in performance are acceptable in our application, because most instances of hand use in an activity of daily living will extend over several seconds, and therefore be reflected in a large number of frames; interpretation of functional activities from the video data should therefore be robust to isolated errors in hand detection or contour identification in individual frames.

Another limitation of the system is that the field of view of the camera used in this study was found to be too small for the current application, such that the hand could easily stray out of the frame. For the purpose of the test videos, care was taken to ensure that the camera was pointed in the right direction at all times, but this was unnaturally constraining. This consideration has no impact on our proposed image processing algorithm, but alternative camera choices should be considered in the future.

The next step in the development of this system, in addition to the improvements suggested above, will be to add a module that can detect interactions of the hand with its environment. Lastly, the system will be implemented on a portable platform (e.g. smartphone) with the goal of performing the image processing in real-time and creating a log of hand use throughout the day (e.g. number of grasp attempts, how many were successful, types of grasps used or objects interacted with, etc.). This information will in turn make it possible for clinical staff to better evaluate the impact of neurorehabilitation interventions in daily life and to adjust the interventions as appropriate. We anticipate that technology developed for this application will also help to create automated scoring procedures for many of the clinical assessments of hand function mentioned in the Introduction, thus improving the reliability and ease of delivery of these measures.

The algorithm proposed here may also be of value to researchers interested in different applications involving wearable video of hand use, beyond the context of neurorehabilitation. Applications in able-bodied individuals could include ergonomics studies investigating manipulation in different environments. Previous studies have also proposed wearable video systems for detecting specific hand gestures [30,36,42], and our method may complement those efforts thanks to its simplicity and flexibility. In particular, an advantage of our method is that it requires no training set, and can therefore be easily implemented by any researchers interested in wearable video of hand activity.

## Conclusion

We have proposed a novel method for identifying the contour of the hand in video obtained from a wearable camera. The two-phase algorithm is applied to each frame in turn and includes: (1) a hand detection and seed point selection step that is based on standard skin colour models; (2) a contour detection step that adaptively identifies an ROI in the colour histogram and creates the corresponding backprojection. The system uses a single commercially available camera, requires no markers, and is fast enough for real-time implementation at modest frame rates. This combination of factors makes it suitable for future clinical use in evaluating neurorehabilitation interventions. To this end, future work will focus on identifying the interactions of the hand with its environment.

## Competing interests

The authors declare that they have no competing interests.

## Authors’ contributions

JZ developed the contour detection algorithms, created the test videos, analyzed the system’s performance, and drafted the manuscript. JZ and MRP both participated in the conception and design of the study. MRP additionally provided critical revision to the manuscript. Both authors read and approved the final manuscript.
